# Interplay Between Sub-Cellular Alterations of Calcium Release and T-Tubular Defects in Cardiac Diseases

**DOI:** 10.3389/fphys.2018.01474

**Published:** 2018-10-25

**Authors:** Marina Scardigli, Cecilia Ferrantini, Claudia Crocini, Francesco S. Pavone, Leonardo Sacconi

**Affiliations:** ^1^National Institute of Optics, National Research Council, Florence, Italy; ^2^European Laboratory for Non-Linear Spectroscopy, Florence, Italy; ^3^Division of Physiology, Department of Experimental and Clinical Medicine, University of Florence, Florence, Italy; ^4^Department of Molecular, Cellular, and Developmental Biology & BioFrontiers Institute, University of Colorado Boulder, Boulder, CO, United States; ^5^Department of Physics and Astronomy, University of Florence, Florence, Italy

**Keywords:** t-tubule, excitation-contraction coupling, calcium imaging, voltage imaging, microscopy

## Abstract

Asynchronous Ca^2+^ release promotes non-homogeneous myofilament activation, leading to mechanical dysfunction, as well as initiation of propagated calcium waves and arrhythmias. Recent advances in microscopy techniques have allowed for optical recordings of local Ca^2+^ fluxes and action potentials from multiple sub-cellular domains within cardiac cells with unprecedented spatial and temporal resolution. Since then, sub-cellular local information of the spatio-temporal relationship between Ca^2+^ release and action potential propagation have been unlocked, providing novel mechanistic insights in cardiac excitation-contraction coupling (ECC). Here, we review the promising perspectives arouse from repeatedly probing Ca^2+^ release at the same sub-cellular location while simultaneously probing multiple locations at the same time within a single cardiac cell. We also compare the results obtained in three different rodent models of cardiac diseases, highlighting disease-specific mechanisms. Slower local Ca^2+^ release has been observed in regions with defective action potential conduction in diseased cardiac cells. Moreover, significant increment of Ca^2+^ variability (both in time and in space) has been found in diseased cardiac cells but does not directly correlate with local electrical defects nor with the degree of structural aberrations of the cellular membrane system, suggesting a role for other players of the ECC machinery. We finally explore exciting opportunities provided by the technology for studying different cardiomyocyte populations, as well as for dissecting the mechanisms responsible for subcellular spatio-temporal variability of Ca^2+^ release.

## Introduction

In cardiac cell, Ca^2+^ release mediates the transduction of an action potential (AP) generated at the cellular membrane (sarcolemma) into contraction of the sarcomeres, in a process called excitation-contraction coupling (ECC) (Bers, 2002). During the AP, Dihydropyridine receptors (DHPR) located on the sarcolemma open, generating an inward Ca^2+^ current (I_caL_), which in turns activate a massive release of Ca^2+^ from the sarcoplasmic reticulum (SR) through ryanodine receptor (RyR) 2 (Kushnir and Marks, 2010). This mechanism is called calcium induced calcium release (CICR) and occurs in the dyads, where RyRs are clustered in release units on the SR juxtaposing DHPRs on the sarcolemma (Scriven et al., 2013). When a DHPR opens, local [Ca^2+^]_i_ rises up to 10–20 μM in less than 1 ms in the junctional cleft and triggers approximately 6–20 RyRs to release at each couplon (Bers, 2001). This increases [Ca^2+^]_i_ in the cleft to 200–400 μM. Ca^2+^ diffuses in the cell as the spatial and temporal summation of each local Ca^2+^ release unit (CRU) (Wier and Balke, 1999) and activates myofilaments that are the end effector of ECC (Bers, 2002). During systole, the strength of twitch depends on SR Ca^2+^ release, which in turn depends on SR Ca^2+^ content and the extent of the Ca^2+^ trigger (Bassani et al., 1995; Hobai and O’Rourke, 2001). After each contraction, [Ca^2+^]_i_ must decrease, so that cardiac cycle is enabled again. There are two main mechanisms to reduce [Ca^2+^]_i_: SR Ca^2+^-ATPase (SERCA) and sarcolemmal Na^+^/ Ca^2+^ exchanger (NCX). The first pumps Ca^2+^ back into the SR consuming ATP, the latter extrudes the cation in the extracellular space, exploiting the electrochemical gradient of Na^+^, with a stoichiometry of 3Na:1Ca (Eisner et al., 1998). This balance between *trans*-sarcolemmal and SR Ca^2+^ influx and efflux is very highly maintained and it is essential for homogeneous myofilaments contraction and relaxation. The two main features of cardiac diseases, such as contractile dysfunction and arrhythmias, are correlated with ECC abnormalities (Ter Keurs and Boyden, 2007). To ensure a homogenous contraction of the entire cardiomyocyte, the AP is rapidly propagated toward the cell core thanks to a complex network of sarcolemmal invaginations named transverse-axial tubular system (TATS) or t-tubules. In control ventricular myocytes containing t-tubules, SR Ca^2+^ release is spatially and temporally synchronized (Cheng et al., 1994; Haddock et al., 1999; Brette et al., 2002, 2004, 2005; Yang et al., 2002).

Ca^2+^ transient desynchronization can be experimentally reproduced in myocytes acutely detubulated through osmotic shock (Brette et al., 2002; Yang et al., 2002) or in cultured myocytes (Louch et al., 2004), mimicking pathological conditions in which t-tubules are disrupted (Kostin et al., 1998; He et al., 2001; Louch et al., 2006; Song et al., 2006; Heinzel et al., 2008; Dibb et al., 2009; Sacconi et al., 2012; Crocini et al., 2016c). The loss of TATS determines a Ca^2+^release initially triggered at sites where t-tubules are present, followed by propagation into regions without TATS, where RyR2 are orphan of their corresponding Ca^2+^ channels (Heinzel et al., 2002).

A common trait of ECC abnormalities consists in the loss of the perfect synchrony between membrane voltage and Ca^2+^ fluxes (Gomez et al., 1997). The interaction between RyR2 and Ca^2+^ channels is finely regulated (Ibrahim et al., 2011) as any spatio-temporal alteration may generate “waves” or “foci” leading to abrupt arrhythmias or, if persisting, to ineffective ECC and severe failure. However, electrical properties of TATS are often studied using indirect or macroscopic observations rather than on direct measurements.

In the last few years, the combination of advanced optical microscopy with novel functional reporters opened the possibility to better understand the role of TATS in both healthy and disease, by unveiling functional features with an unprecedented spatio-temporal resolution. In this regard, our group has recently developed an ultrafast random access two-photon microscope (Sacconi et al., 2012). Combining this technology with novel fluorinated voltage sensitive dyes with improved photostability (Yan et al., 2012) and calcium probes, we have simultaneously measured action potentials (APs) and intracellular calcium release at multiple sites within the TATS with sub-millisecond temporal and sub-micrometer spatial resolution. In the present work, we will review the capability of this method in providing important insights on sub-cellular alterations of calcium release and in dissecting their relationship with TATS morpho-functional defects found in different cardiac diseases.

## Sub-Cellular Recordings of Calcium Release and Action Potentials

**FIGURE 1:**
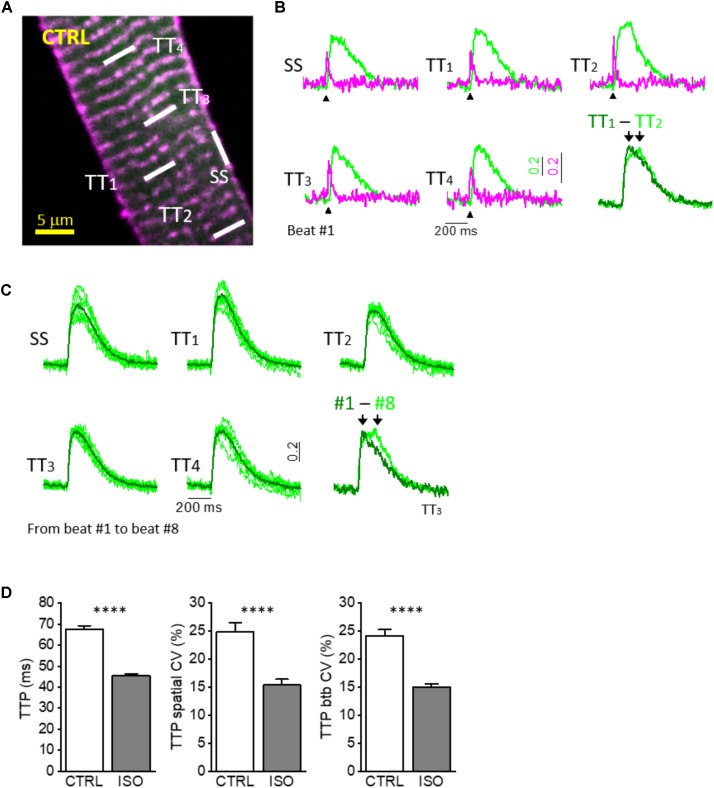
Simultaneous multisite voltage and Ca^2+^ recordings. **(A)** Two-photon fluorescence (TPF) image of a stained rat ventricular myocyte: sarcolemma in magenta (di-4-ANE(F)PTEA) and [Ca^2+^]_i_ in green (GFP-certified Fluoforte). Modified from Crocini et al. (2014b). **(B)** Normalized fluorescence traces (ΔF/F_0_) simultaneously recorded from the scanned sites indicated in white in **A**: surface sarcolemma (SS) and four T-tubules (TTi). AP is elicited at 200 ms (black arrowheads). Membrane voltage (magenta) and [Ca^2+^]_i_ (green). The last is a superimposition of two simultaneous Ca^2+^ transients. Modified from Crocini et al. (2014b). **(C)** Superimposition of ten subsequent Ca^2+^ transients recorded from five scanned sites in a CTRL cardiomyocyte. The last is a superimposition of one scanned t-tubule obtained from different subsequent beating (recording #1 and recording #8). **(D)** Graphs showing mean values for Ca^2+^ transient time-to-peak (TTP), time-to-peak spatial variability coefficient (TTP spatial CV), and time-to-peak beat-to-beat variability coefficient (TTP btb CV) of Ca^2+^ release. Data reported as mean ± SEM from 122 TTs (27 CTRL cells from 10 rats) and 48 TTs (11 ISO cells from 3 rats). Student’s *t*-test applied, ^∗∗∗∗^*P* < 0.0001.

In the last few decades, several techniques have been developed in order to get functional information of the TATS in isolated cardiomyocytes. In particular, optical methodologies have met the need to reduce invasiveness of traditional methods and have undergone a wide evolution. The major limiting factor to optically record fast physiological events, like AP and Ca^2+^ release in multiple site of the same cardiomyocyte, has been the scanning time that restricts recordings to only a single position of the cell at the time, i.e., line scanning approach. A strategy to overcome this limitation could consist in spending more time just to collect as many photons as possible from selected positions. We have recently implemented a new technique based on Random Access Multiphoton (RAMP) (Iyer et al., 2006) microscopy to record AP propagation and Ca^2+^ release at different domains of the same cardiomyocyte in a simultaneous fashion (Sacconi et al., 2012; Crocini et al., 2014a,b, 2016c). In detail, the RAMP microscope is a two-photon microscope provided with an ultrafast scanning head made of two orthogonal acousto-optic deflectors (AODs) that diffract a laser beam at a precise angle, which can be changed within a few microseconds. Based on the velocity of sound propagation in the crystal present in the AODs and the dimension of the laser beam, a commutation time of about 4 μs can be estimated between a scanning line and the next in multiplexed modality. In order to get information about the two main actors of ECC, isolated cardiomyocytes from rodents were stained with FluoForte GFP-certified, a fluorescent Ca^2+^ indicator, and di-4-AN(F)EPPTEA, a fluorinated voltage-sensitive dye (VSD) (Yan et al., 2012): the RAMP microscope was used to simultaneously excite both dyes and, thanks to the large Stokes shift of the fluorinated VSD, the two fluorescence signals were easily distinguished using appropriate optical tools. Figures 1A,B illustrates the real-time simultaneous optical recording of AP and Ca^2+^ transients in isolated cardiomyocytes at different non-contiguous domains. Isolated cardiac cells were field stimulated at room temperature (Sacconi et al., 2012; Crocini et al., 2014b) to reach steady state. Then, we performed real-time simultaneous optical recordings consisting of 10 consecutive recordings, where we probed 5–10 different tubular membrane sites per recording (TTi, *i* = 1 to 10) (Figure 1C). The length of scanning lines for each membrane site ranged from 2 to 10 μm with an integration time of 200 μs per line, leading to a temporal resolution of 0.4–2 ms. The signal-to-noise ratio (S/N) was sufficient to detect the presence of an AP occurring at sarcolemma and to assess the temporal features of the Ca^2+^ transient in the surrounding cytoplasm. Local calcium transients at each TTi were analyzed in terms of amplitude and kinetics and we calculated a coefficient of variability (CV) as the ratio between the standard deviation and the mean of a specific parameter, namely the time to peak of Ca^2+^ release.

Of note, by averaging the 10 consecutive RAMP measurements performed at each site, we have demonstrated the tight electrical coupling between surface sarcolemma (SS) and t-tubules (TTs) (Sacconi et al., 2012), as well as the perfect overlap of Ca^2+^ transients originating close to SS and TTs during steady-state stimulations in control ventricular cardiomyocytes (Crocini et al., 2014b). These results have demonstrated the uniformity of Ca^2+^ release across the entire cardiomyocyte and highlighted the crucial role of the TATS in propagating AP into the whole cell ensuring a synchronous Ca^2+^ release. Exploiting the possibility to simultaneously and consecutively perform several measurements of selected regions, we obtained information regarding Ca^2+^ release variability in both space and time. These two parameters appeared to be of peculiar interest:

1. The spatial CV of time to peak (spatial CV of TTP), that is the ratio of the standard deviation to the mean between TTi = 1 to 10 recorded during a single activation (e.g., trial 1 or trial 2 or trial 3, etc.).
2. The beat-to-beat CV of time to peak (btb CV of TTP), that is the ratio of the standard deviation to the mean between consecutive activations (trial 1, trial 2, trial 3 etc.) calculated at a specific membrane site (e.g., TT1 or TT2 or TT3, etc.).

Regarding the mechanisms underlying the observed spatio-temporal variability, we first hypothesized that the presence of a non-negligible spatial CV (approximately 25%; Figures 1C,D) between far apart TT_i_ in control rat ventricular cardiomyocytes could be related to heterogeneous functional or structural characteristics at different calcium release sites. Non-homogeneous phosphorylation levels of RYR2 or DHPRs in space, for instance, or variability in the dyadic cleft dimensions/structure could lead to a large spatial CV even in control cells. This hypothesis was denied when we observed a quantitatively similar variability of Ca^2+^ release in time, i.e., when we compared consecutive activations at the same single t-tubule site. In control rat ventricular cardiomyocytes btb CV of TTP was also in the order of 25% (Figures 1C,D) On a beat-to-beat basis, no functional or structural changes at the calcium release sites may occur that could account for a relatively large (25%) CV and, in our view, the sole possible mechanism underlying the phenomenon is the stochastic nature of Ca^2+^ release itself, that our RAMP technique appears able to detect. Indeed, only a few Ca^2+^ release units (CRUs) reside within the volume probed by each scanned line (5–10 CRUs in ∼10 μm^3^) (Franzini-Armstrong et al., 1999; Soeller et al., 2007). A typical CRU hosts about 50–200 RyRs and 10–25 Ca^2+^ release channels (Franzini-Armstrong et al., 1999). During normal twitch activation (at low inotropic levels) only a small fraction of RyR2 is recruited (e.g., of the total ∼3 million of RyR2 in the cell, less than 2–5% would open during a regular twitch) (Wier et al., 1994; Bers, 2001). This low open probability (Po) of the RyR2 explains the high variability of Ca^2+^ release that we observed. We expected that by increasing RyR2 Po the spatial and btb CV of TTP would decrease. In order to enhance RyR2 Po, we performed measurements in the presence of a β-adrenergic agonist, isoproterenol (ISO, 10^-7^ M), that acutely promotes RyR2 hyperphosphorylation at Protein Kinase A (PKA) sites, thus prolonging the duration of RyR2 opening (RyR2 t_on_) and enhancing RyR2 cytosolic and luminal Ca^2+^ sensitivity (Eisner et al., 2004; Ferrantini et al., 2016). As expected, we found that both spatial and btb CV of TTP were markedly reduced under ISO: the extent of the reduction (Figure 1D) was similar for the two parameters. This result suggests that the local variability of TTP (in space and time) is tightly related to the functional state of RyR2s and reflects the stochastic nature of their openings. Of note, to some extent, the synchronization of local Ca^2+^ release under ISO could be attributed to a more intense trigger (i.e., slower I_CaL_ inactivation), and further experiments with a direct RyR2 agonist would be helpful to define the reciprocal role of the two mechanisms (Ca^2+^ channels *vs*. RyR2 PKA-mediated phosphorylation).

## T-Tubule Structural Remodeling and Calcium Alterations in Cardiac Diseases

The ability of our method to probe the spatiotemporal relationship between Ca^2+^ and electrical activity was then explored in three models of cardiac disease characterized by TATS morpho-functional remodeling.

All animal handling and procedures were performed in accordance with the guidelines from Directive 2010/63/EU of the European Parliament on the protection of animals used for scientific purposes and conformed to the principles and regulations as described in the editorial by Grundy (2015). The experimental protocol was approved by the Italian Ministry of Health (protocol number 647/2015-PR).

We compared the following pathological settings:

• a rat model of ischemic heart failure (HF-ischemic), in which myocardial infarction was induced by ligation of the left anterior coronary artery as previously described (Lyon et al., 2009); the animals were studied 8–10 weeks after the ischemic damage, when echocardiographic signs of heart failure were (such as left ventricle (LV) dilation and reduced LV contractility) easily revealed.
• a rat model of left ventricular hypertrophy induced by spontaneous hypertension (i.e., spontaneously hypertensive rats, SHR). Notably we analyzed the late-stage of the disease, when LV dilation and failure occurred. Thus, this model represents a second example of overt heart failure (HF-SHR) (Okamoto and Aoki, 1963).
• a mouse model of hypertrophic cardiomyopathy (HCM), expressing a human mutated cardiac troponin T (deletion of a codon at position 160 of the protein, cTnT Δ160E), associated with high risk of sudden cardiac death and severe diastolic dysfunction in patients (Pasquale et al., 2012; Coppini et al., 2014). Mice employed were 6–8 months old, reflecting early stages of the disease, when LV function is compensated (Moore et al., 2013).

**FIGURE 2:**
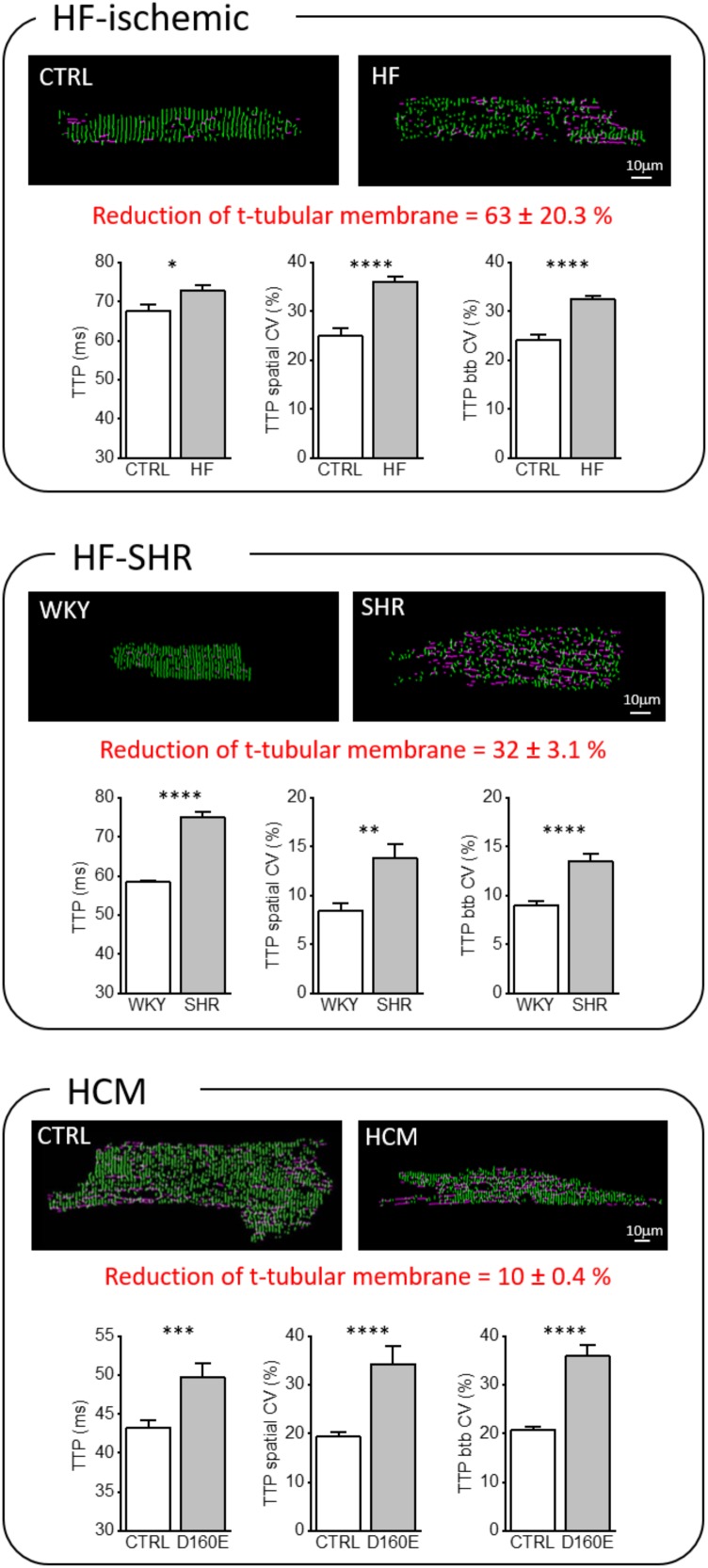
Transverse-axial tubular system architecture and Ca^2+^ kinetics in ischemic heart failure (HF-ischemic), spontaneously hypertensive rats in overt heart failure (HF-SHR), and hypertrophic cardiomyopathy (HCM). On the top, representative images of cellsshowing skeletonized T-tubular system, obtained with AUTO-TT. Transverse elements are shown in green and axial ones in magenta. On the bottom, Graphs showing mean values for Ca^2+^ transient time-to-peak (TTP), time-to-peak spatial variability coefficient (TTP spatial CV), and time-to-peak beat-to-beat variability coefficient (TTP btb CV) of Ca^2+^ release. Data reported as mean ± SEM from 122 TTs (27 CTRL cells from 10 rats), 364 TTs (59 HF cells from 12 rats), 87 TTs (25 WKY cells from 8 rats), 87 TTs (23 SHR cells from 8 rats), 101 TTs (28 CTRL cells from 10 mice), and 66 TTs (17 Δ160E cells from 7 mice). Auto TT images of: HF-SHR from Scardigli et al. (2017); of CTRL and HCM from Crocini et al. (2016c). Student’s *t*-test applied, ^∗^*P* < 0.05, ^∗∗^*P* < 0.01, ^∗∗∗^*P* < 0.001, ^∗∗∗∗^*P* < 0.0001.

In order to quantify the loss of TATS transverse components (TT) from confocal images of isolated cardiomyocytes, we used AutoTT, a software developed specifically for quantitative analysis of the TATS architecture (Guo and Song, 2014). Cardiomyocytes isolated from the three pathological models introduced above showed different levels of t-tubular remodeling (Figure 2). In particular, we found a variable reduction of TT density, more accentuated in the HF-ischemic model (HF-ischemic > HF-SHR > HCM). Disruption of TT in failing cardiomyocyte was previously reported in a number of animal models and in human myocytes (He et al., 2001; Song et al., 2006). In a rat model of left ventricular hypertrophy, the loss of t-tubules was described as a marker of the transition from compensated hypertrophy to HF (Wei et al., 2010). Our genetic-based model of LV hypertrophy (HCM) demonstrates that t-tubule disruption can occur also during the compensated stages of LV hypertrophy (Crocini et al., 2016c), highlighting the “detubulation” process as an early event of cardiomyocyte remodeling.

Most of the studies regarding membrane architecture in pathological conditions have been conducted with lipophilic membrane-selective fluorescent dyes and reported a reduction of t-tubule staining. Two distinct possibilities underlie this observation. First, t-tubules may seal off from the surface membrane and remain as (permanent or transitory) sealed-off intracellular membrane structures. Alternatively, t-tubules could be truly depleted from the “diseased” cells with a real loss of membrane domains and associated proteins. Discrimination between these possibilities would be crucial to acquire new insights about pathological t-tubular turnover, but rather difficult to explore experimentally. We instead focused our attention on the function of the “residual” remodeled TATS elements. Besides a reduction of transverse components, remodeled TATS show: (1) a reduction in the number of t-tubular openings on the surface (mouths) (Wagner et al., 2012), (2) a greater proportion of tubules running in the longitudinal and oblique directions (Louch et al., 2006; Swift et al., 2008), (3) increased mean t-tubular diameter and length (Maron et al., 1975; Kaprielian et al., 2000), and (4) tubular proliferation, with enhanced t-tubular tortuosity, increased number of constrictions and branches (Kostin et al., 1998). These geometrical changes, characterized with nanometric resolution by STED microscopy (Wagner et al., 2012; Kohl et al., 2013), go hand in hand with structural alterations of the dyadic cleft, as well as changes of membrane channels density and function. In failing myocytes, an expansion of the dyadic cleft occurs, resulting in a greater distance between Ca^2+^ channels and RyR2 (Gomez et al., 1997; Wei et al., 2010; van Oort et al., 2011). Recently, an increased variability in dyadic cleft distance has been also described (Wei et al., 2010). There is a general consensus that I_CaL_ density is unchanged in HF and HCM cardiomyocytes when measured during voltage-clamp steps (Bers, 2006; Coppini et al., 2013), but the number of Ca^2+^channels is reportedly reduced (He et al., 2001). Increased single Ca^2+^channel activity appears to maintain normal Ca^2+^ current density in HF (Schroder et al., 1998), and this may result from increased phosphorylation of the Ca^2+^channel by PKA and/or Ca^2+^/calmodulin kinase II (CaMKII) (Chen et al., 2002). Similarly, RyR2 density in HF and HCM cardiomyocytes appear to be unchanged (Gomez et al., 2001; Benitah et al., 2002; Coppini et al., 2013) but channel function is enhanced by post-translational modifications. For instance, chronic “hyper-phosphorylation” of RyR2 by PKA (Marx et al., 2000) and CaMKII (Ai et al., 2005; Curran et al., 2010; Grimm et al., 2015; Pereira et al., 2017) that occurs in HF causes dissociation of FKBP12.6 from RyR2, destabilizes the RyR2 complex and causes functional uncoupling of neighboring RyRs.

In addition to phosphorylation (Marx et al., 2000; Braz et al., 2004; Wehrens et al., 2004), RYR2 is regulated by other post-translational modifications such as oxidation and S-nitrosylation (Xu et al., 1998; Eu et al., 2000; Barouch et al., 2002; Santos et al., 2011) as well as accessory and structural proteins (e.g., triadin, junctin, and junctophilin-2) (van Oort et al., 2011). Every level of RyR2 regulation can be impaired in HF (Yano et al., 2005), with the functional effect of destabilizing RYR2 closed states.

Thus, while arrays of RyR2s normally tend to open and close together, RyR2 functional uncoupling in failing myocytes is proposed to result in a greater open probability during diastole and desynchronised channels opening during systole. Increased dyadic cleft distance associated with impaired Ca^2+^ channels and, more importantly, impaired RyR2 function in the CRUs may lead to a general disruption of local Ca^2+^ release synchronicity in remodeled TATS.

Using the RAMP microscope, we have directly addressed this issue and observed that a number of functional abnormalities are common in “residual” TATS of HF-ischemic, HF-SHR, and HCM cardiomyocytes: Ca^2+^ transient TTPs are prolonged and both the spatial and btb CV (of TTPs) are increased (Figure 2). We believe that the structural TATS and dyadic changes combined with the impaired RyR2 regulation described above play a major role in the increased local variability of TTP observed in the three diseases. However, exploiting the possibility of RAMP technique to simultaneously map local TATS electrical activity and measure corresponding Ca^2+^ fluxes, we found that the increased TTPs could be also correlated to electrical abnormalities occurring at the single t-tubular level (Sacconi et al., 2012; Crocini et al., 2014b; 2016c; Scardigli et al., 2017).

## Relationship Between Electrical T-Tubular Defects and Local Alterations of Calcium Release

**FIGURE 3:**
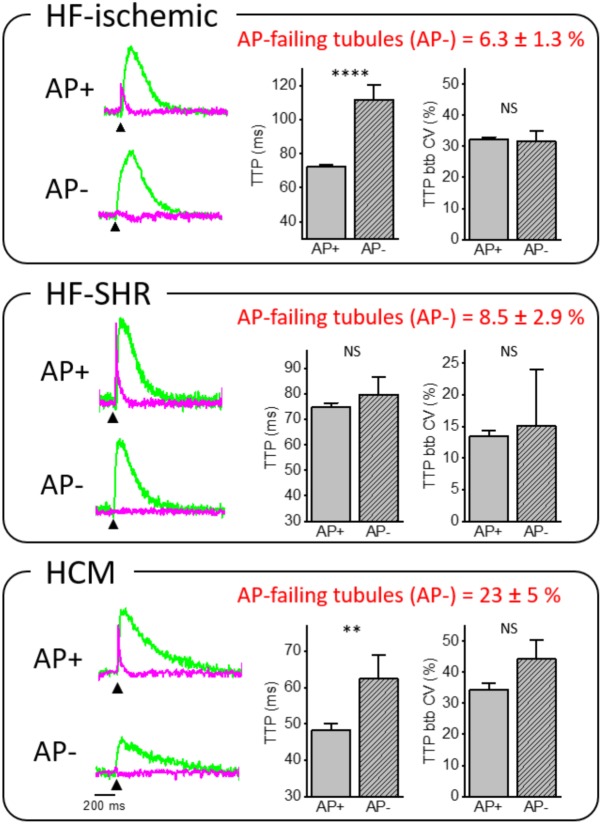
Relationship between electrical defects and Ca^2+^ kinetics in ischemic heart failure (HF-ischemic), spontaneously hypertensive rats in overt heart failure (HF-SHR), and hypertrophic cardiomyopathy (HCM). On the left, normalized fluorescence traces (ΔF/F_0_) of voltage (magenta) and [Ca^2+^]_i_ (green) recorded from the two scanned sites. An action potential (elicited at 200 ms, black arrowheads) is clearly visible in AP+ but absent in AP– within the same cell. On the right, graphs showing mean values for Ca^2+^ transient time-to-peak (TTP) and time-to-peak beat-to-beat variability coefficient (TTP btb CV) of Ca^2+^ release in AP+ and AP–. Data reported as mean ± SEM from 341 AP+ and 23 AP– (59 HF cells from 12 rats), 80 AP+ and 7 AP– (23 SHR cells from 8 rats), and 51 AP+ and 15 AP– (17 Δ160E cells from 7 mice). Traces of HCM from Crocini et al. (2016c) Student’s *t*-test applied, NS *P* > 0.05, ^∗∗^*P* < 0.01, ^∗∗∗∗^*P* < 0.0001.

Our laboratory has demonstrated that loss of the TATS leads to alteration of AP propagation. A number of t-tubules fail to propagate AP from the surface sarcolemma into the cell core (failing T-tubule; AP-) (Figure 3) in diseased cardiomyocytes. In two different rat models of heart failure, HF-ischemic and HF-SHR, (6.3 ± 1.3)% and (8.5 ± 2.9)% of t-tubules, respectively, were not able to conduct the AP, while a regular stimulated AP occured on the surface sarcolemma and neighbor t-tubules. Interestingly, in cTnT Δ160E ventricular cardiomyocytes, we observed (23 ± 5)% of AP-failing t-tubules, while TATS morphological alterations were minimal (Crocini et al., 2016c). This result suggests that the number of failing t-tubules is not correlated with the degree of lost t-tubular elements, but is due to local ultrastructural alterations (Crocini et al., 2016b; Scardigli et al., 2017). Further support comes from our findings regarding acutely detubulated cardiomyocytes, in which a dramatic detachment of t-tubules was associated with only (12 ± 4)% of failing t-tubules among the remaining connected elements (Sacconi et al., 2012). Based on the pathological substrate, electrical defects may profoundly impair local Ca^2+^ release determining significant differences of Ca^2+^ release TTP between coupled (AP+) and uncoupled (AP-) t-tubules. Ca^2+^ transient TTPs were significantly prolonged in AP- as compared to corresponding AP+ in HF-ischemic and HCM myocytes. These results strongly suggest that the Ca^2+^ transient detected at AP- tubules originates from the Ca^2+^ signal propagating from neighboring electrically coupled sites (AP+). This phenomenon was confirmed by a follow-up work, in which we employed a β-adrenergic agonist in HF-ischemic myocytes (Crocini et al., 2016a). At variance with AP+ tubules, Ca^2+^ transient TTPs at AP- sites were not affected by β-adrenergic stimulation, validating the propagative Ca^2+^-cascade. In fact, β-adrenergic signaling does not regulate the velocity of Ca^2+^ propagation in the cell.

Interestingly, btb CV of TTP was not affected by the electrical coupling of the corresponding t-tubule in all of the disease settings we have investigated. Importantly, this result indicates that local Ca^2+^ release variability measured with our technique is determined by RyR2 functional state rather than propagative Ca^2+^-cascade from functioning AP+ sites to AP-. Again, this result has been confirmed by acute treatment of HF-ischemic myocytes with β-adrenergic agonist (Crocini et al., 2016a), in which btb CV of TTP was reduced upon β-adrenergic stimulation. We can conclude that β-adrenergic signaling can regulate RyR2 functional states, resulting in a reduced CV in any location of the cell. Differently from HF-ischemic and HCM, Ca^2+^ kinetics did not show a statistical difference between AP+ and AP- in SHR/HF cardiomyocytes. This result remains of difficult interpretation and may be related to the pronounced delay of the overall Ca^2+^ transient that involves the whole cell in SHR (Bing et al., 1991).

## Conclusion and Prespectives

We employed the RAMP microscope to simultaneously and consecutively measure local TATS activity and obtain information regarding Ca^2+^ release kinetics and variability of Ca^2+^ transient time to peak both in space (spatial CV) and in time (btb CV). We found that, in control cardiomyocytes, space and btb CV of TTP are similar one other (approximately ∼25%) and are both reduced by half upon isoproterenol treatment. Starting from the consideration that a relative small number of RyR2 are located in the scanned regions, our results in control cardiomyocytes suggest that the local variability of TTP may be tightly related to the stochastic opening of RyR2s and is influenced by the channel functional state. The beat-to-beat variability at single membrane domains strongly exclude the presence of systematic structural or functional factors underlying the variability in space. The stochastic nature of Ca^2+^ release is further confirmed by the fact that mean local Ca^2+^ transients, resulting from averaging 10 consecutively measurements, perfectly overlap in terms of amplitude and kinetics between SS and TT. In our view, the fact that an average “in time” abolishes the spatial variability is a further demonstration that the mechanisms underlying this variability is the stochasticity of Ca^2+^ release.

We next studied the electrical function and associated Ca^2+^ fluxes of “residual” structurally remodeled TATS in three pathological conditions (HF-ischemic, HF-SHR, HCM) and found a number of common changes as well as disease-specific alterations: prolonged local Ca^2+^ transients TTPs and markedly increased spatial and temporal variability of TTPs. Despite different numbers, we found a population of “residual” t-tubules failing to propagating AP in all three pathological settings. We found two main results. The first (expected) was an increased spatial and btb CV of TTPs in disease remodeled TATS. The second (unexpected) was that the electrical coupling of t-tubules (AP+ and AP-) does not influence the variability of Ca^2+^ release.

At glance, the spatial CV appears to be the more relevant parameter in terms of functional implications for non-homogenous sarcomere activation and the generation of pro-arrhythmogenic triggers through mechano-electric feedback. In diseased myocardium, where t-tubules are structurally and functionally remodeled and the spatial variability of Ca^2+^ release is increased, the coexistence of “in parallel” myofibril layers with different timing of initial activation would result in reduced force development and overall slower kinetics. However, we can speculate that the increased temporal variability of Ca^2+^ release may generate also the coexistence of “in series” myofibrils with different timing of activation, eventually promoting arrhythmias by favoring AP alternans. These new pro-arrhythmogenic mechanisms that occur in remodeled t-tubules may be added to those previously described (Orchard et al., 2013; Crocini et al., 2014a).

A number of mechanisms may contribute to the increased spatial and btb CV of TTPs of remodeled TATS, including dyadic structural changes and alterations of Ca^2+^ channels density and regulation. However, the absence of difference in btb CV of TTPs between AP+ and AP-, conditions where local I_CaL_ triggers are present (AP+) and absent (AP-), respectively, exclude a major role of Ca^2+^ channels function and indicates that the main player in the local variability of Ca^2+^ release is the RyR2 functional state. While RyR2 function resulting from targeted single post-translational modification (e.g., the effects of single phosphorylation at specific sites) has been largely studied in channels re-expressed in bilayers, the complex RyR2 alterations that occur in cardiac diseases cannot be reproduced *in vitro*. Preserving the manifold context of pathological settings is therefore essential to accurately investigate disease-related channel alternations. Thanks to our RAMP approach, a novel set of information may become accessible, allowing for unprecedented exploration of RyR2 function in living cardiomyocytes. Distinct modulation of different subpopulations of RyR2 clusters, those coupled to the t-tubules (dyadic RyR2) and those non-coupled (corbular RyR2), has been recently highlighted in a number of studies (Dries et al., 2013, 2016). CaMKII-dependent potentiation of RyR2 activity, for instance, is restricted to coupled RyRs in the dyadic cleft (Dries et al., 2013), while PKA phosphorylation occur at all RyRs concurrently, including those “non-coupled” to the sarcolemmal membrane (Dries et al., 2013, 2016). In addition to the subcellular microdomain compartmentalization, the phosphorylations by CaMKII and PKA differently affect RyR2 gating as well as its modulation by a number of factors (e.g., cytosolic and luminal calcium, regulatory proteins) (Bers, 2001). As an example, the mean open probability (Po) is increased by both kinases, but the effects on channel open times (To) and channel closed times (Tc) are specific and may directly affect the stochasticity of Ca^2+^ release and its subcellular spatio-temporal variability. The scenario of RyR2 subpopulations at different microdomains is even more complex in cells that exhibit a t-tubular network poor of transverse elements but enriched of other components (e.g., axial tubules, AT).

In the absence of abundant transverse membrane invaginations, as in atrial cells or in disease-remodeled ventricular cardiomyocytes, we can configure the presence of at list other three populations of RyR2 in addition to those mentioned above:

- RyR2s that are coupled to axial tubules, well represented in atrial cells.

- RyR2s that are “orphaned” of their t-tubules because of t-tubule disruption in disease-associated structural remodeling.

- RyR2s that are coupled to non-propagating (AP-) t-tubules, as observed in disease-remodeled myocytes and described in detail above.

The study of subcellular spatio-temporal variability of Ca^2+^ release with the RAMP technique may provide significant insights to clarify the functional characteristics of these RyR2 populations. For instance, in mouse atrial myocytes, Ca^2+^ release from the SR in axial tubules is approximately two times faster at the center of the cell as compared to Ca^2+^ release at the surface. Rapid Ca^2+^ release correlated with colocalization of highly phosphorylated (highphos) RyR2 clusters at junctions between axial tubules and SR junctions (Brandenburg et al., 2016). Novel evidence of atrial “super-hub” Ca^2+^ signaling has been recently reported across different species (Brandenburg et al., 2018), possibly establishing a new paradigm for atrial Ca^2+^ release. We can speculate that the spatio-temporal variability of Ca^2+^ release could be reduced at the highphos RyR2 clusters and our approach may help establishing the role of axial tubules, highphos RyR2 clusters, and non-junctional Ca^2+^ release. RAMP measurements can be extended to the intact tissue in order to study subcellular Ca^2+^ release spatio-temporal variability in various cells at the same time (Sacconi et al., 2012; Ferrantini et al., 2014). These studies could include human myocardium, whose characteristics are only partly reproduced by murine models, thus providing an even further interesting perspective to dissect RyR2 function in a physiological environment. In fact, recent studies in failing human hearts agree on the reduction of t-tubule density, which was two to three times lower in failing ventricular myocytes from HF patients with different aetiologies than in healthy donors (Cannell et al., 2006; Lyon et al., 2009). Loss of t-tubules have been also described in patients with genetic-based cardiomyopathies (Maron et al., 1975; Kaprielian et al., 2000; Ferrantini et al., 2018). We believe that our studies on rodent models of HF and congenital cardiac diseases could (qualitatively) reflect the spatio-temporal variability of Ca^2+^ release that may occur in human cardiomyocytes. Though, direct RAMP measurements of Ca^2+^ release in human t-tubules are needed, and may represent the most exciting perspective for this technology.

## Author Contributions

MS analyzed the data. MS, CC, CF, FP, and LS wrote the paper.

## Conflict of Interest Statement

The authors declare that the research was conducted in the absence of any commercial or financial relationships that could be construed as a potential conflict of interest.The handling Editor declared a shared affiliation, though no other collaboration, with several of the authors CF, FP, and LS at the time of review.
